# Supplementary material for: Tau filaments with the Alzheimer fold in human MAPT mutants V337M and R406W
Source: Nat Struct Mol Biol. 2025 Mar 5;32(7):1297–304. doi: 10.1038/s41594-025-01498-5 (PMC12263442; doi:10.1038/s41594-025-01498-5)
Supplement: Supplementary file 1 — Reporting Summary [file 41594_2025_1498_MOESM1_ESM.pdf]

nature portfolio

Corresponding author(s): Michel Goedert

Last updated by author(s): Dec 22, 2024

Reporting Summary

Nature Portfolio wishes to improve the reproducibility of the work that we publish. This form provides structure for consistency and transparency in reporting. For further information on Nature Portfolio policies, see our Editorial Policies and the Editorial Policy Checklist.

Statistics

For all statistical analyses, confirm that the following items are present in the figure legend, table legend, main text, or Methods section.

n/a	Confirmed	
☐	☒	The exact sample size (*n*) for each experimental group/condition, given as a discrete number and unit of measurement
☐	☒	A statement on whether measurements were taken from distinct samples or whether the same sample was measured repeatedly
☒	☐	The statistical test(s) used AND whether they are one- or two-sided<br>*Only common tests should be described solely by name; describe more complex techniques in the Methods section.*
☒	☐	A description of all covariates tested
☒	☐	A description of any assumptions or corrections, such as tests of normality and adjustment for multiple comparisons
☐	☒	A full description of the statistical parameters including central tendency (e.g. means) or other basic estimates (e.g. regression coefficient) AND variation (e.g. standard deviation) or associated estimates of uncertainty (e.g. confidence intervals)
☒	☐	For null hypothesis testing, the test statistic (e.g. *F*, *t*, *r*) with confidence intervals, effect sizes, degrees of freedom and *P* value noted<br>*Give P values as exact values whenever suitable.*
☒	☐	For Bayesian analysis, information on the choice of priors and Markov chain Monte Carlo settings
☒	☐	For hierarchical and complex designs, identification of the appropriate level for tests and full reporting of outcomes
☒	☐	Estimates of effect sizes (e.g. Cohen's *d*, Pearson's *r*), indicating how they were calculated

Our web collection on statistics for biologists contains articles on many of the points above.

Software and code

Policy information about availability of computer code

Data collection	EPU2.3.079 (thermofisher scientific)
Data analysis	Relion4, ctffind4.1,chimera1.18, servalcat, refmac5, ISOLDE, MolProbity4.5, COOT 0.9.8.7, ChimeraX 1.6.1

For manuscripts utilizing custom algorithms or software that are central to the research but not yet described in published literature, software must be made available to editors and reviewers. We strongly encourage code deposition in a community repository (e.g. GitHub). See the Nature Portfolio guidelines for submitting code & software for further information.

Data

Policy information about availability of data

All manuscripts must include a data availability statement. This statement should provide the following information, where applicable:

- Accession codes, unique identifiers, or web links for publicly available datasets
- A description of any restrictions on data availability
- For clinical datasets or third party data, please ensure that the statement adheres to our policy

Cryo-EM maps have been deposited in the Electron Microscopy Data Bank (EMDB) with accession numbers: EMD-19846; EMD-19849; EMD-19852; EMD-19854; EMD-19855. Corresponding refined atomic models have been deposited in the Protein Data Bank (PDB) under the following accession numbers: 9EO7; 9EO9; 9EOE; 9EOG; 9EOH.

Research involving human participants, their data, or biological material

Policy information about studies with human participants or human data. See also policy information about sex, gender (identity/presentation), and sexual orientation and race, ethnicity and racism.

Reporting on sex and gender	see method section. 3 cases of Seattle family with mutation V337M, sex: female, famale, male. 1 case US family with mutation R406W, sex:female. 1 case UK family with mutation R406W, sex:male.
Reporting on race, ethnicity, or other socially relevant groupings	Not relevant to study.
Population characteristics	see method section. 3 cases of Seattle family with mutation V337M, death age 78,63, 58. 1 case US family with mutation R406W, death age 78. 1 case UK family with mutation R406W, death age 66.
Recruitment	Selected based on neuropathological examination.
Ethics oversight	For the V337M MAPT cases, informed consent for brain donation was obtained from the legal next of kin according to protocols approved by the University of Washington Institutional Review Board that conform to the provisions of the Declaration of Helsinki and preserve donor anonymity. For the R406W MAPT case 1, research protocols for the Indiana Alzheimer's Disease Research Center were approved by the Indiana University Institutional Review Board (protocol:1011003338, initial approval date:04/22/1991, current expiration date:02/05/2025). Brain tissue from R406W MAPT case 2 was donated to the UCL Queen Square Brain Bank with informed consent and the study was approved by the NHS Health Research Authority Ethics Committee, London-Central (REC reference: 23/LO/0044). Genomic DNAs from the V337M and R406W cases were extracted from postmortem brain tissues. The cryo-EM study was approved by the Cambridgeshire Research Ethics committee (09/HO308/163).

Note that full information on the approval of the study protocol must also be provided in the manuscript.

Field-specific reporting

Please select the one below that is the best fit for your research. If you are not sure, read the appropriate sections before making your selection.

☒ Life sciences ☐ Behavioural & social sciences ☐ Ecological, evolutionary & environmental sciences

For a reference copy of the document with all sections, see nature.com/documents/nr-reporting-summary-flat.pdf

Life sciences study design

All studies must disclose on these points even when the disclosure is negative.

Sample size	We used frontal cortex from three previously described cases of Seattle family A with mutation V337M in MAPT. We used temporal and parietal cortex, as well as hippocampus from a female with mutation R406W in MAPT (US family). We used frontal, temporal and parietal cortices from a male with mutation R406W in MAPT (UK family).
Data exclusions	No data excluded.
Replication	All attempts at replication were successful.
Randomization	Because there is no assignment of data points to distinct groups, randomization was not applicable to this study.
Blinding	No blinding was performed, as the risk for bias by the experimentalist was deemed irrelevant for this study.

Reporting for specific materials, systems and methods

We require information from authors about some types of materials, experimental systems and methods used in many studies. Here, indicate whether each material, system or method listed is relevant to your study. If you are not sure if a list item applies to your research, read the appropriate section before selecting a response.

Materials & experimental systems

n/a	Involved in the study
☐	☒ Antibodies
☒	☐ Eukaryotic cell lines
☒	☐ Palaeontology and archaeology
☒	☐ Animals and other organisms
☒	☐ Clinical data
☒	☐ Dual use research of concern
☒	☐ Plants

Methods

n/a	Involved in the study
☒	☐ ChIP-seq
☒	☐ Flow cytometry
☒	☐ MRI-based neuroimaging

Antibodies

Antibodies used

Primary antibodies used are presented in the Methods section.
BR134 (Diluted 1:1000) (made by crb Camrbidge Research Biochemicals as request, against human tau C-terminus)
AT8 (Diluted 1:1000 or 1:300)(thermofisher scientifc, Catalog # MN1020)
RD3 (Diluted 1:3000) (Milipore 05-803, culture supernatant, clone 8E6/C11, Upstate®)
RD4 (Diluted 1:100) ((Milipore 05-804,clone 1E1/A6)
anti-4R (Diluted 1:400)(Cosmo Bio Catalog No:CAC-TIP-4RT-P01)

Validation

BR134 validated against human tau C-terminus in (Goedert et al. 1989 Neuron 3,519-526)
AT8 validated against human tau pS202 and pT205 in manufacturer's datasheet (Thermofisher scientific). This Antibody was verified by Cell treatment to ensure that the antibody binds to the antigen stated).
RD3 is validated against human 3R tau in manufacturer's datasheet (Millipore)
RD4 is validated for use in IH, WB for the detection of Tau (4-repeat isoform RD4).
Anti-4R validated against human tau residues 275-291 in (Falcon et al. 2018 Nature 561,137-140) and validated for western blot and IHC(p), this isoform-specific tau antibody is useful for immunohistochemical and biochemical studies of tau species in diverse neurodegenerative diseases. (Cosmo Bio)

Plants

Seed stocks

Not relevant to study.

Novel plant genotypes

Not relevant to study.

Authentication

Not relevant to study.
