# Supplementary figures and images for: Tau filaments with the Alzheimer fold in human MAPT mutants V337M and R406W
Source: Nat Struct Mol Biol. 2025 Mar 5;32(7):1297–304. doi: 10.1038/s41594-025-01498-5 (PMC12263442; doi:10.1038/s41594-025-01498-5)

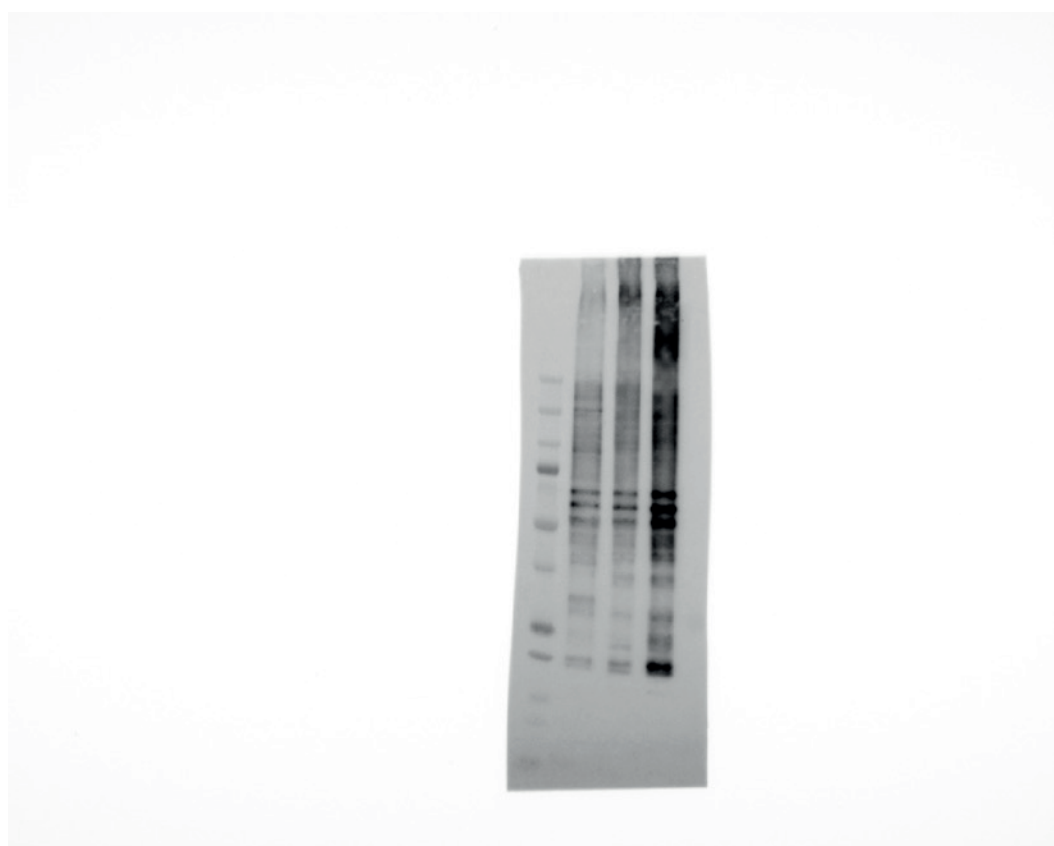

Supplement: Supplementary file 3 — Unprocessed western blots. [file 41594_2025_1498_MOESM3_ESM.pdf]

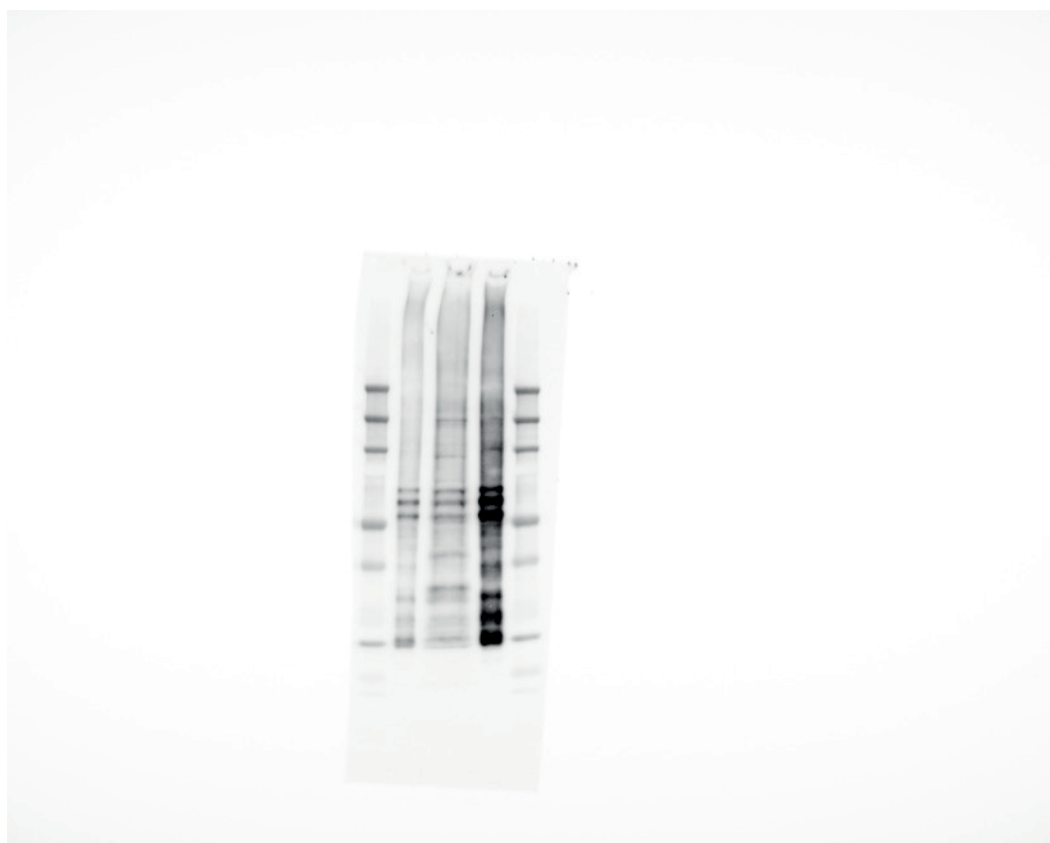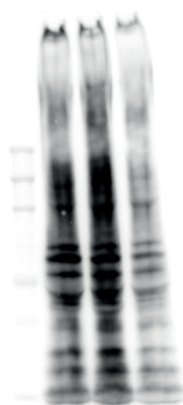

Supplement: Supplementary file 5 — Unprocessed western blots. [file 41594_2025_1498_MOESM5_ESM.pdf]

R406W Case 1 Parietal cortex

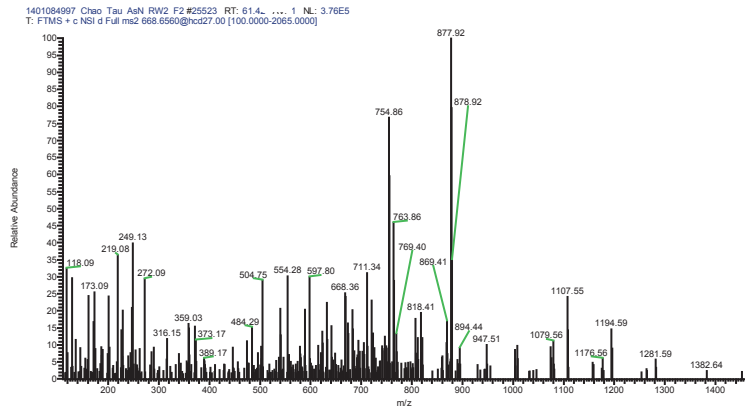

R406W Case 1 Parietal cortex

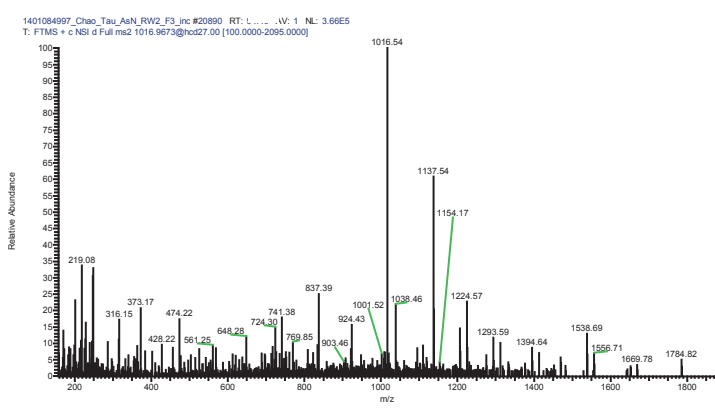

R406W Case 2 Frontal cortex

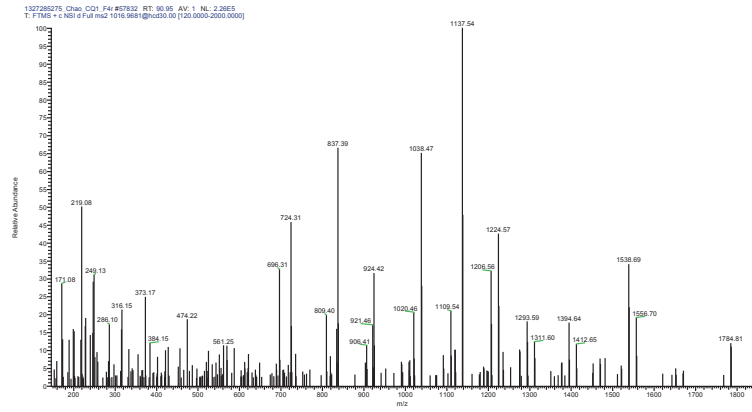

Supplement: Supplementary file 7 — Unprocessed MS. [file 41594_2025_1498_MOESM7_ESM.pdf]
