# Supplementary material for: Tau filaments with the Alzheimer fold in human MAPT mutants V337M and R406W
Source: Nat Struct Mol Biol. 2025 Mar 5;32(7):1297–304. doi: 10.1038/s41594-025-01498-5 (PMC12263442; doi:10.1038/s41594-025-01498-5)

V337M Case 1 Frontal cortex

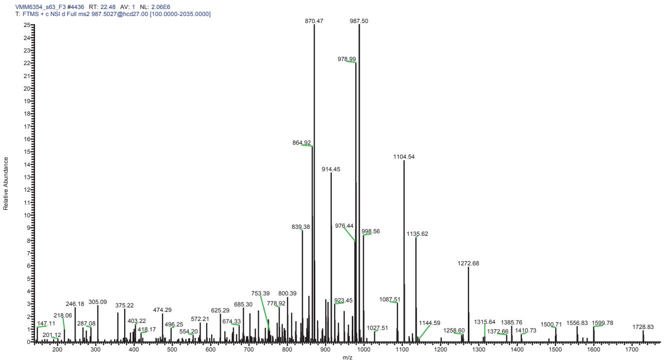

V337M Case 1 Frontal cortex

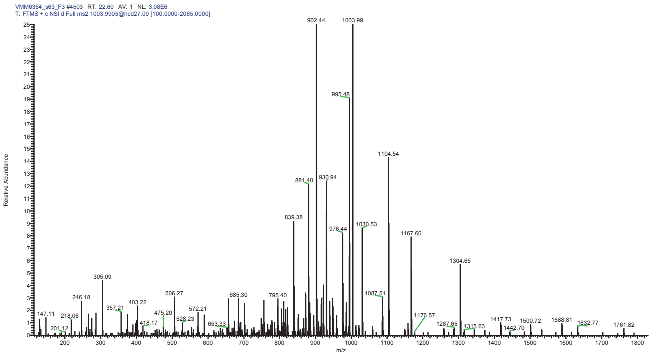

V337M Case 2 Frontal cortex

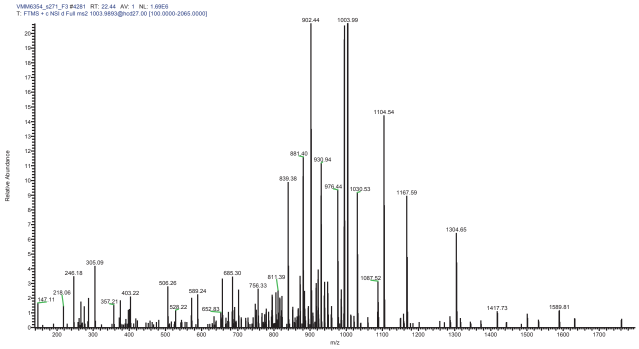

V337M Case 2 Frontal cortex

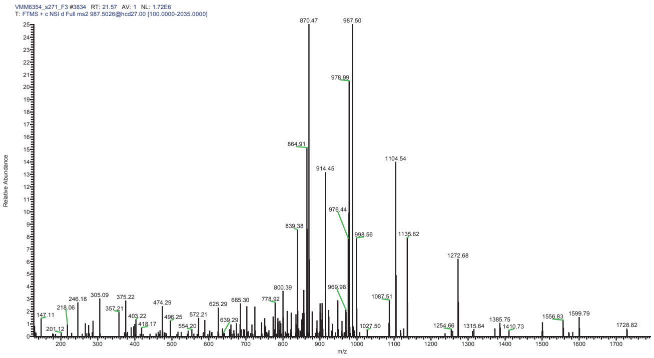

V337M Case 3 Frontal cortex

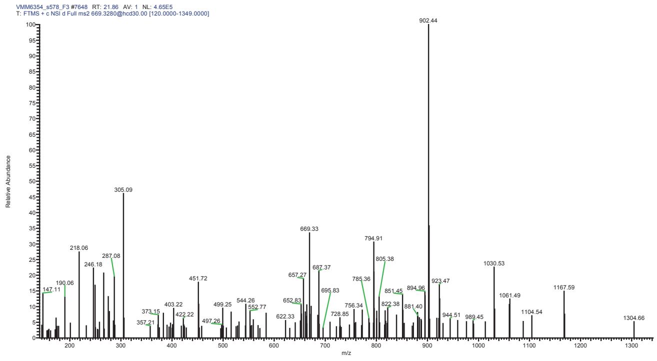

V337M Case 3 Frontal cortex

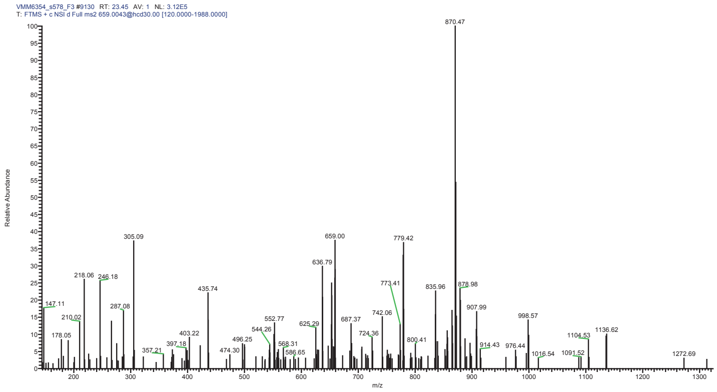

Supplement: Supplementary file 6 — Unprocessed MS. [file 41594_2025_1498_MOESM6_ESM.pdf]
